# How Does Background Music Affect Dining Duration, Tips and Bill Amounts in Restaurants? A Field Experiment

**DOI:** 10.3390/bs14121188

**Published:** 2024-12-13

**Authors:** Merav Malcman, Ofer H. Azar, Tal Shavit, Mosi Rosenboim

**Affiliations:** 1 Faculty of Management and Economics, Ruppin Academic Center, Hefer Valley 40250, Israel; meravm@ruppin.ac.il; 2 The Department of Business Administration, Ben-Gurion University of the Negev, Beer Sheva 84105, Israel; 3 The Department of Economics and Business Administration, Ariel University, Ariel 40700, Israel; talsh@ariel.ac.il; 4 The Department of Management, Ben-Gurion University of the Negev, Beer Sheva 84105, Israel; mmm@bgu.ac.il

**Keywords:** music tempo, restaurants, tips, field experiment, background music

## Abstract

Influences from external factors can affect decision-makers, preventing them from making decisions in a fully rational manner. Music may serve as one such influential factor in this context. Music is part of our daily lives, and we are exposed to music in numerous places. We designed a field experiment to study the influence of background music on patrons’ behavior in restaurants. Specifically, we examine the effect of the music’s tempo (slow or fast) on time spent in the restaurant, the bill amount, and the tip size. The results show that patrons in the slow tempo group spent the most time in the restaurant, those in the control group followed next, and the patrons in the fast tempo group were the quickest to leave. However, there are no differences between the groups in bill size. The tips in the fast tempo group were higher than in the control group when controlling for several independent variables. The findings have practical implications for restaurant owners and managers. In busy periods, the restaurant can use fast-tempo music to increase the turnover of tables, because then tables become available for new diners more quickly, contributing more to the restaurant’s income compared to tables that occupy the space for a longer duration.

## 1. Introduction

Completely rational economic decisions might not happen as often as we think [1]. Occasionally, the decision-making is interrupted by interventions (nudges, external elements, environmental factors, etc.) that affect the effectiveness of the decisions and the decision process [2]. The literature in behavioral economics shows that factors in our environment can affect our decisions even if these factors seem irrelevant. This shows that economic choices are only sometimes completely logical and can be influenced by external elements. How we decide can also be influenced by how information is presented [3].

One of the environmental factors is the background music that surrounds us. Music is part of our daily lives, and we are exposed to music almost everywhere: at home or at work, at the mall or in a restaurant or bar [4,5,6,7]. Although music should not affect the cost or utility of consuming services or products, the literature suggests that music can influence people’s decision-making in a variety of situations [8,9,10,11]. Specifically, some studies have found that people react to music in everyday commercial environments (e.g., restaurants, markets, wine stores, etc.). The review by Palazzi et al. [12] shows that music is a powerful and engaging stimulus that influences decision-making processes and affects customers’ behavioral choices.

Demoulin [13] demonstrates that music congruent with the atmosphere in services cape (a physical environment in which a service is delivered) creates relaxed, calm, and pleasant feelings. Music also creates an atmosphere in tourism settings [14] and is perceived by customers in terms of pleasure and arousal [15]. Music also influences a customer’s mood [16], and customers who are in an elevated mood evaluate the service environment and service quality more positively. The influence of background music has also been examined in everyday decision-making, such as in financial decisions where it has been found that fast-tempo music leads people to make riskier decisions [17,18].

Spence [19] shows that background music influences the atmospheric elements in retail settings. In a meta-analysis study, Kampfe et al. [20] found that the effects of background music in retail settings are not always apparent and are not uniform across individuals when they do occur. Some studies suggest that music is used to prime consumer behavior and purchase decisions [21,22,23], with the effect of the music depending on the nature of the activity and the characteristics of the background music. North et al. [24] concluded that background music in a supermarket influences the purchase decision. Areni and Kim [21] showed that classical music encourages purchases of expensive wine. In a laboratory setting, Fiegel et al. [25] found that participants liked the food they were eating more when the music was pleasant and stimulating. Specifically, music with a faster tempo enhanced the flavor of bell peppers relative to silence. Ziv [26] showed that cookies tasted better with pleasant music in the background than in a condition with unpleasant music.

Only a few field studies have been carried out to determine the effect of music in commercial settings and, in particular, in restaurants. Some studies have focused on the way music influences a customer’s evaluation of the sounds made when eating (such as chewing sounds) [5,19,25]. Biswas et al. [5] demonstrated that soft (low volume) background music in restaurants encourages the choice of healthy food, in contrast to high-volume music or no music. Guéguen et al. [27] showed that high music volume increased alcohol consumption and reduced the average amount of time spent by the patrons to drink. The order in which products are presented also played a role: the first product presented against a background of pleasant music was rated as tasting better [5]. Furthermore, it has been found that background music in a restaurant that customers perceive to be congruent with the atmosphere (as they define it), leads to a more pleasant dining experience [13]. Likewise, background music consisting of “drinking” songs about good food and alcohol (such as those sung on festive days) causes customers in bars to stay longer and drink more (where the control is top 40 hits or songs from animated films; [6]).

The field experiment we carried out examined the effect of the background music’s tempo on patrons’ behavior in a restaurant. The literature suggests that tempo is one of the most important determinants of human response to music [28]. Gagnon and Peretz [29] found that tempo influences emotional judgment, and Kampfe et al. [20] found that tempo is strongly correlated with arousal and has a clear effect on behavior. Fast music has been shown to raise listeners’ self-reported arousal levels [8,30,31]. Similarly, Ding and Lin [9]. show that the tempo of background music raises the arousal of online shoppers. Dixon et al. [32] found that in a casino a fast tempo leads to bets being placed more quickly relative to a slow tempo or no music and Buelow et al. [33] show that when music played in the background during testing, no significant differences in task performance were found, but the music was associated with riskier decisions compared to no music condition. McElrea and Standing [34] show that fast music significantly decreased drinking time.

Research on music tempo in consumer behavior shows that fast music increases purchase intentions more than slow music [35]. Tempo also affects time perception [31,36,37], such that a slow tempo leads patrons to underestimate time spent in the restaurant. From a healthy lifestyle perspective, slower music tempo extended eating duration, which led to consuming less food [38].

Since tempo is the element of music most commonly found to influence customer behavior, previous research in restaurants has focused on that. Slow music led customers to spend more time in the restaurant [36,37,39] and to spend more money on alcohol [37]. Caldwell and Hibbert [36,39] also found that it led to more spending on food and beverages.

In contrast to most past experiments which were based on self-reporting by customers, in our experiment we measure their actual behavior. Furthermore, we have adopted a somewhat different methodology. Thus, for example, Milliman [37] collected the data from a restaurant only on Fridays and Saturdays and ignored the number of diners at a table. It also manipulated the music selection so that the tempo was unambiguously identifiable. In contrast, we collect data on various days of the week and limit the size of the party to two adults (in order to avoid peer influences). Furthermore, we did not change the restaurant’s usual playlist. Another example is Caldwell and Hibbert [36,39] who collected data on Thursdays and Sundays (in order to avoid the effect of waiting outside to get in, which occurs on Fridays and Saturdays). Furthermore, only jazz music was used and only that of a single artist. Finally, they collected the data from just two specific tables at the restaurant and prior to leaving the restaurant, the patrons were given a questionnaire which asked about, among other things, the perceived time they had spent in the restaurant. In our study, the patrons were unaware they were in an experiment, they did not answer a questionnaire, data were gathered from all tables and the music was the same as that normally heard in the restaurant (with the only difference being in the tempo).

While Caldwell and Hibbert [36,39] collected data via the aforementioned questionnaire, which included variables such as perceived time spent in the restaurant, enjoyment of the meal, intention to return and intention to recommend, only a few studies have included the amount of the bill. Furthermore, and to the best of our knowledge, no prior study has examined the effect of music on the tip amount. Tipping is an important economic phenomenon with a variety of motivations and implications [40,41,42,43,44,45]. The main reasons that customers tip are to avoid violations of social norms, to avoid feelings of embarrassment and guilt, the awareness that waiters depend on tips for their income, and the desire to show gratitude [42].

The rest of the paper is organized as follows: Section 2 presents the hypotheses. Section 3 describes the experimental design. Section 4 presents the results, and Section 5 provides a conclusion.

## 2. Working Hypotheses

Music is recognized as a significant environmental factor in shaping consumer experiences and behaviors. In restaurant settings, background music contributes to creating an ambiance that can influence patrons’ behavior. The tempo of music plays a critical role in regulating arousal levels and mood, and is easier to identify, measure and modify than other structural features [46], which are known to affect behaviors such as time spent, spending money, and prosocial actions like tipping. This study aims to deepen our understanding of how music tempo affects customer behavior in restaurants, and, in particular, how it affects time spent, bill amounts, and tipping.

As mentioned, Milliman [37] found that the tempo of background music has an effect on time spent in a restaurant and increases the consumption of alcohol. Caldwell and Hibbert [39] also found that slow music led customers to spend more time in the restaurant. Therefore, the first hypothesis is as follows:

**H1:**  *When the tempo of the music is slower the customers will spend more time in the restaurant.*

Caldwell and Hibbert [36] found that a slow tempo increases the amount spent by customers. Therefore, the second hypothesis is as follows:

**H2:**  *When the tempo of the music is slower, the bill amount will be higher.*

It has been found that the music’s tempo influences the mood of the patrons [39,47], and that fast-tempo music is more likely to induce a positive mood [29,48]. Moreover, previous research shows that a positive or negative mood will increase helping behavior relative to a neutral mood [49,50]. Therefore, we hypothesize that the music’s tempo may affect tipping behavior, since music’s tempo, as an environmental factor, is known to influence mood and arousal levels [39], which in turn can affect prosocial behaviors, such as tipping. Tipping is a prosocial act that helps others, because it sacrifices money that the tipper has and transfers it to others (usually the worker who provided the service, unless several workers split the tips), without a legal obligation to do so. It therefore shares characteristics of making donations, where the donors also transfer money voluntarily to others. Fast-paced music tends to increase arousal, which can lead to heightened emotional states such as excitement. These elevated states can make individuals more willing to engage in behaviors that align with those heightened emotions, such as generosity or tipping. Moreover, social norms and expectations often govern tipping behavior in restaurants [41]. Fast music, by increasing arousal and mood, might make patrons more aware of social norms related to generosity. Therefore, our third hypothesis is as follows:

**H3:**  *Tips will be higher when the tempo of the music is faster.*

## 3. Experimental Design and Methodology

### 3.1. Manipulations

The two treatment conditions were created based on the criteria used by Milliman [37] and reused by Caldwell and Hibbert [36,39]. In a preliminary survey, Milliman [37] asked the restaurant’s patrons to classify the background music’s tempo. Based on the responses, it was decided that music with 94 or more beats per minute would be considered a fast tempo, while 72 or fewer beats per minute would be considered a slow tempo. This classification was used to establish the treatment groups of fast tempo and slow tempo in the current experiment. The background music for the control group consisted of the restaurant’s regular playlist, which was a mix of both slow and fast tempo songs. When the data were collected, the music tempo manipulation remained constant throughout the entire shift. This ensured that in the two manipulation treatments, patrons were exposed exclusively to either fast-tempo or slow-tempo music during their visit. Volume was similar in all the manipulations.

The restaurant’s regular playlist in the experiment was a combination of well-known and lesser-known female and male vocalists. A company that specializes in constructing playlists for background music in restaurants, and with whom the restaurant has an ongoing contract, selected songs from the restaurant’s regular playlist of Italian and Greek music for each of the manipulations, according to the specified tempo classification. They thus created two playlists for the manipulations: one with fast tempo and the other with slow tempo. The music was played using the shuffle option on the restaurant’s regular sound system.

### 3.2. Data Collection and Experimental Procedures

The experiment was carried out in an Italian restaurant located in central Tel Aviv, with the permission of the restaurant’s management (the restaurant is open seven days a week and is very busy all week; it has been in business for approximately 20 years, serving a clientele that is mostly middle class and above). Different days were randomly divided into control and experimental conditions and the music on each day throughout the restaurant was based on the condition chosen for that day. This ensured that there is no systematic difference between the conditions in terms of which day of the week they occur on. The assignment of days to conditions also achieved a balanced number of participants across the experimental groups. The participants were unaware that they were part of an experiment. The control group heard the regular playlist, whereas the experimental groups were manipulated using two tempo conditions: slow and fast. Because this was a field experiment, patrons were not given a questionnaire, and data were collected exclusively from field observations. The experiment was approved by the ethics committee of the University.

The data were collected over a period of several months in the evening hours from tables with parties of 1–2 adults, to eliminate peer effects on decision-making. Overall, data were collected from 282 tables. One of the authors was present during the data collection. This author sat at a side table in a position that allowed a clear view of the participants involved in the experiment. Additionally, this author had access to the restaurant’s computer system to collect data on bill and tip amounts through the payment system. The waiters reported tip amount when it was a cash tip and additional variables (e.g., time of arrival, time of paying the bill, bill amount, credit card tips) were collected either by the author who was present or from the restaurant’s system. Bill and tip amounts are for the entire table and are denominated in ILS (with ILS 1 equivalent to about USD 0.27 at the time of the experiment).

## 4. Results

Table 1 reports the descriptive statistics for the two music manipulations and the control group.

Tempo had a statistically significant effect on time spent in the restaurant. The average time spent in the restaurant in minutes was lower in the fast music group than in the slow music group (t = −7.811, *p* < 0.01) and the control group (t = −4.227, *p* < 0.01). As shown in Table 1, patrons in the slow music group spent an average of 80.3 min in the restaurant, 40.2% longer than the fast tempo group who stayed an average of 57.29 min. The average time for the control group was 69.22 min, 20.8% longer than the fast tempo group. Finally, the slow tempo group spent 16% more time in the restaurant than the control group (80.3 vs. 69.22 min), and that difference is also statistically significant (t = 3.103, *p* < 0.01).

Table 2 reports the results of a regression analysis with two different dependent variables: Model (1)—time spent in the restaurant; Model (2)—tip amount. Table 3 further reports the results of a regression analysis of the dependent variable bill amount, where Model (3) includes the variable Time, and in Model (4), the variable time is dropped. The independent variables are the following:

(1) FastTempo—a dummy variable for fast tempo (1 for fast tempo and 0 otherwise).
(2) SlowTempo—a dummy variable for slow tempo (1 for slow tempo and 0 otherwise).
(3) Alcohol—a dummy variable for the consumption of alcohol during dinner (1 for alcohol consumed and 0 otherwise).
(4) BillCredit—the method used to pay the bill (1 for credit card and 0 for cash).
(5) TipCredit—the method used to pay the tip (1 for credit card and 0 for cash).
(6) Time—the number of minutes the customers spent in the restaurant (from the time of arrival until paying the bill).
(7) Bill amount (total bill for the table) and tip amount are in ILS.

Model (1) shows that a slow tempo has a significantly positive effect on time spent in the restaurant, and that a fast tempo has a significantly negative effect on it. Both effects are consistent with H1. Model (2) shows that a fast tempo has a significantly positive effect on the tip amount, thus supporting H3. In addition, and not surprisingly, the bill amount has a significantly positive effect on the tip amount.

Model (3) shows that the consumption of alcohol and time spent in the restaurant have a significantly positive effect on the bill amount. H2 is not supported because the music tempo does not affect the bill amount. Moreover, Model (4) shows that also without the time variable, the regression still yields the same insignificant result for the music tempo, further reinforcing the conclusion that music tempo does not affect the bill amount.

## 5. Conclusions

Past research on the influence of background music on human behavior has had mixed results. Kampfe et al. [20] meta-analysis study showed that the effect of background music is context-dependent, there are situations in which music has no effect, and the effect of music is not uniform in magnitude. Behne [51] claimed that since people are exposed to so much music in their everyday lives, they are no longer susceptible to the influence of background music. Trompeta et al. [52] carried out a meta-analysis of music in tourism and hospitality settings which showed that the design of the music rather than its presence is what influences customers. One of their findings is that music tempo does not significantly affect total expenditure or consumption, length of stay or evaluation of the music.

The results of our experiment indicate that a slow music tempo increases time spent in the restaurant (longer time). However, music tempo does not influence the bill amount, and this contrasts with previous studies that found that the effect of music tempo on the bill amount is statistically significant [39,53]. Another interesting finding relates to the tip amount: fast music tempo has a positive effect on the tip amount relative to the control group. It is possible that the fast music tempo leads the customer to act more impulsively and thus leave a larger tip. Another possible explanation is that the fast music tempo caused the customers to perceive that they had received a fast service which was deserving of a larger tip. Finally, consistent with previous literature showing that music tempo influences mood and arousal levels, the fast music tempo may put the customer in a good mood which can also lead to a larger tip.

The current study is novel in several ways. First, the sample is larger compared to previous studies (282 tables as compared to about 60 patrons or less). Furthermore, we also used a control group (which heard the regular playlist). The data for the control variables in the regression analysis were observed in the field, rather than being based on responses to a questionnaire. We also considered variables such as the tips that were not addressed before, and the sample is from a country (Israel) that was not analyzed previously in this context.

There are nonetheless some limitations to the study. Since this was a field experiment and participants were unaware that they were part of an experiment, it was not possible to collect data on subjective variables or to ask participants about their reactions to the music tempo. This was necessary in order to ensure natural behavior of customers. Lab experiments may be able to analyze additional variables by asking subjects questions, but at the cost of no longer observing natural behavior in a real-world setting.

In addition, the study was conducted at a single restaurant in one country. Future research conducted across more restaurants and in multiple countries could extend the understanding of the factors that affect the influence of music on restaurant diners’ behavior. For example, the restaurant in our experiment was an Italian restaurant and it could be interesting to explore whether in other types of restaurants the effect of music tempo may be different. In addition, two additional music components, namely mode and genre, could impact diners’ behavior, and future research can address their impact.

This study contributes to our understanding of the effect of music on consumer behavior in restaurants. A practical implication of the findings is that fast-tempo music can be used to increase a restaurant’s efficiency and profitability. Thus, if a restaurant is crowded, fast-tempo background music can be used to increase the turnover of tables, making tables available for new diners more promptly and increasing income. Fast-tempo music can also be used to achieve larger tips. On the other hand, restaurant managers could adjust the playlist to include slower-tempo music during less busy shifts. This strategy could help extend patrons’ stay time, and create the illusion of a fuller restaurant, which may be more pleasant for the diners and beneficial for the restaurant’s reputation when there are fewer customers present.

The results may also be applicable to additional environments other than restaurants. They suggest that people stay longer when they hear slow-tempo music and stay a shorter time when they hear fast-tempo music. This could have practical implications for various businesses. For example, a shopping mall that wants to encourage people to stay longer (and thus to make more purchases) should use slow-tempo music. On the other hand, an amusement park that wants visitors to shorten their staying time (to be able to accommodate additional customers and to avoid long queues) may want to use fast-tempo music.

## Figures and Tables

**Table 1:** Descriptive statistics.

	Number of Tables	Mean	STD	Min	Max
**Fast music**	99				
Time in minutes		57.29	14.98	15	120
Tip percentage (The ratio of tip amount to bill amount)		14.57%	3.29%	6.38%	29.87%
Bill amount		197.31	69.07	62	432
Tip amount		28.71	12.37	8	90
**Slow music**	96				
Time in minutes		80.3	25.04	25	171
Tip percentage		14.06%	3.12%	7.89%	25%
Bill amount		207.36	68.41	92	410
Tip amount		29.32	12.58	9	90
**Control group**	87				
Time in minutes		69.22	23.08	31	176
Tip percentage		13.65%	3.53%	0%	28.90%
Bill amount		208.79	72.17	72	474
Tip amount		28.02	10.90	0	76
**All treatments**	282				
Time in minutes		68.80	23.35	15	176
Tip percentage		14.11%	3.35%	0%	29.87%
Bill amount		204.28	69.76	62	474
Tip amount		28.71	11.98	0	90

**Table 2:** Linear regression results: time and tip amount as dependent variables.

	Model (1) Time			Model (2) Tip Amount		
Independent Variable	B	SE B	*p*-Value	B	SE B	*p*-Value
Time				−0.004	0.020	0.853
Bill amount	0.045 *	0.020	0.027	0.140 **	0.007	0.000
FastTempo	−11.398 **	3.145	0.000	2.219 *	1.049	0.035
SlowTempo	11.352 **	3.190	0.000	1.574	1.064	0.140
Alcohol	−3.460	2.887	0.232	0.224	0.943	0.813
BillCredit	−0.792	3.532	0.823	−0.651	1.151	0.572
TipCredit	2.807	3.364	0.405	−0.264	1.098	0.810
Constant	60.431	4.824	0.000	−0.592		0.764
F	10.439 **			80.394 **		
Adjusted R^2^	0.168			0.664		

Comments: * stands for *p*-value < 0.05; ** stands for *p*-value < 0.01. The *p*-values are two-tailed.

**Table 3:** Linear regression results: bill as a dependent variable.

Dependent Variable	Model (3) Bill Amount			Model (4) Bill Amount		
Independent Variable	B	SE B	*p*-Value	B	SE B	*p*-Value
Time	0.389 *	0.175	0.027	----	----	----
FastTempo	−3.743	9.431	0.692	−8.324	9.268	0.370
SlowTempo	−4.302	9.558	0.653	0.117	9.415	0.990
Alcohol	63.180 **	7.578	0.000	62.944 **	7.631	0.000
BillCredit	−3.807	10.347	0.713	−4.189	10.419	0.688
TipCredit	−2.401	9.869	0.808	−1.332	9.927	0.893
Constant	157.179	14.966	0.000	183.932	8.948	0.000
F	12.653 **			13.997 **		
Adjusted R^2^	0.199			0.188		

Comments: * stands for *p*-value < 0.05; ** stands for *p*-value < 0.01. The *p*-values are two-tailed.

## Data Availability

The data file will be provided by the authors upon request by email.
